# Supplementary material for: COZOID: contact zone identifier for visual analysis of protein-protein interactions
Source: BMC Bioinformatics. 2018 Apr 6;19:125. doi: 10.1186/s12859-018-2113-6 (PMC5889581; doi:10.1186/s12859-018-2113-6)
Supplement: Supplementary file 2 — Software build. Executable binary file of the software tool. (ZIP 118,784 kb) [file 12859_2018_2113_MOESM2_ESM.zip › cozoid/java/docs/beansbinding-1.2.1-doc.zip!/org/jdesktop/el/class-use/MethodNotFoundException.html]

Uses of Class org.jdesktop.el.MethodNotFoundException (Beans Binding)


---


|  |  |  |
| --- | --- | --- |
| **Overview** \| **Package** \| **Class** \| **Use** \| **Tree** \| **Deprecated** \| **Index** \| **Help** | |  |
| PREV   NEXT | **FRAMES**    **NO FRAMES**     **All Classes** |  |


---


## **Uses of Class org.jdesktop.el.MethodNotFoundException**

| Packages that use MethodNotFoundException | |
| --- | --- |
| **org.jdesktop.el.impl** | Customized version of EL for Beans Binding; not for general use. |
| **org.jdesktop.el.impl.util** | Customized version of EL for Beans Binding; not for general use. |

Uses of MethodNotFoundException in org.jdesktop.el.impl

| Methods in org.jdesktop.el.impl that throw MethodNotFoundException | |
| --- | --- |
| `MethodInfo` | `MethodExpressionImpl.getMethodInfo(ELContext context)`             Evaluates the expression relative to the provided context, and returns information about the actual referenced method. |
| `java.lang.Object` | `MethodExpressionImpl.invoke(ELContext context, java.lang.Object[] params)`             Evaluates the expression relative to the provided context, invokes the method that was found using the supplied parameters, and returns the result of the method invocation. |

Uses of MethodNotFoundException in org.jdesktop.el.impl.util

| Methods in org.jdesktop.el.impl.util that throw MethodNotFoundException | |
| --- | --- |
| `static java.lang.reflect.Method` | `ReflectionUtil.getMethod(java.lang.Object base, java.lang.Object property, java.lang.Class[] paramTypes)`             Returns a method based on the criteria |

---


|  |  |  |
| --- | --- | --- |
| **Overview** \| **Package** \| **Class** \| **Use** \| **Tree** \| **Deprecated** \| **Index** \| **Help** | |  |
| PREV   NEXT | **FRAMES**    **NO FRAMES**     **All Classes** |  |


---
